# Myocardial remodelling in aortic regurgitation: time to think beyond volumes and function?

**DOI:** 10.1093/ehjci/jeaf264

**Published:** 2025-09-05

**Authors:** George D Thornton, Michael McKenna, Jonathan B Bennett, Alun Hughes, Arantxa González, Mohammed Y Khanji, João L Cavalcante, Guy Lloyd, James C Moon, Sanjeev Bhattacharyya, Thomas A Treibel

**Affiliations:** Barts Heart Centre, St Bartholomew’s Hospital, West Smithfield, London EC1A 7BE, UK; Institute of Cardiovascular Science, University College London, 62 Huntley St, London WC1E 6DD, UK; Barts Heart Centre, St Bartholomew’s Hospital, West Smithfield, London EC1A 7BE, UK; Department of Cardiology, St Vincent’s University Hospital, Elm Park, Dublin 4, Ireland; Barts Heart Centre, St Bartholomew’s Hospital, West Smithfield, London EC1A 7BE, UK; Institute of Cardiovascular Science, University College London, 62 Huntley St, London WC1E 6DD, UK; Institute of Cardiovascular Science, University College London, 62 Huntley St, London WC1E 6DD, UK; Program of Cardiovascular Disease, CIMA Universidad de Navarra and IdiSNA, Pamplona, Spain; CIBERCV, Institute of Health Carlos III, Madrid, Spain; Barts Heart Centre, St Bartholomew’s Hospital, West Smithfield, London EC1A 7BE, UK; Queen Mary University, University of London, London, UK; Department of Cardiology, Newham University Hospital, London, UK; Department of Cardiology, Allina Health Minneapolis Heart Institute, Minneapolis, USA; Barts Heart Centre, St Bartholomew’s Hospital, West Smithfield, London EC1A 7BE, UK; Queen Mary University, University of London, London, UK; Barts Heart Centre, St Bartholomew’s Hospital, West Smithfield, London EC1A 7BE, UK; Institute of Cardiovascular Science, University College London, 62 Huntley St, London WC1E 6DD, UK; Barts Heart Centre, St Bartholomew’s Hospital, West Smithfield, London EC1A 7BE, UK; Institute of Cardiovascular Science, University College London, 62 Huntley St, London WC1E 6DD, UK; Barts Heart Centre, St Bartholomew’s Hospital, West Smithfield, London EC1A 7BE, UK; Institute of Cardiovascular Science, University College London, 62 Huntley St, London WC1E 6DD, UK

**Keywords:** valvular heart disease, MRI, aortic valve disease, aortic regurgitation

## Abstract

Current guideline criteria for surgical intervention in chronic aortic regurgitation (AR) rely on fixed thresholds of left ventricular size and ejection fraction, but these metrics may overlook early myocardial injury and under-appreciate patient heterogeneity, particularly in women and older adults. Cardiovascular magnetic resonance (CMR) offers robust quantification of regurgitant volume, three-dimensional ventricular volumes, and both focal (late gadolinium enhancement) and diffuse (T1-mapping–derived extracellular volume) fibrosis. Observational studies have linked CMR-detected fibrosis to worse clinical outcomes and less favourable reverse remodelling after valve intervention, suggesting that fibrosis may mark the transition from compensated overload to irreversible myocardial damage. In this narrative review, we appraise the limitations of current guidelines, compare echocardiographic and CMR approaches to AR assessment, and summarize the evidence supporting myocardial fibrosis as a potential imaging biomarker for risk stratification. We discuss how integrating CMR-derived fibrosis metrics with volumetric and functional data could personalize timing of aortic valve intervention. While prospective studies are needed to validate fibrosis-guided decision-making, this evolving paradigm holds promise for earlier identification of patients at risk for irreversible myocardial injury, with the ultimate goal of preserving ventricular function and improving long-term outcomes.

## Graphical Abstract

**Graphical Abstract:**
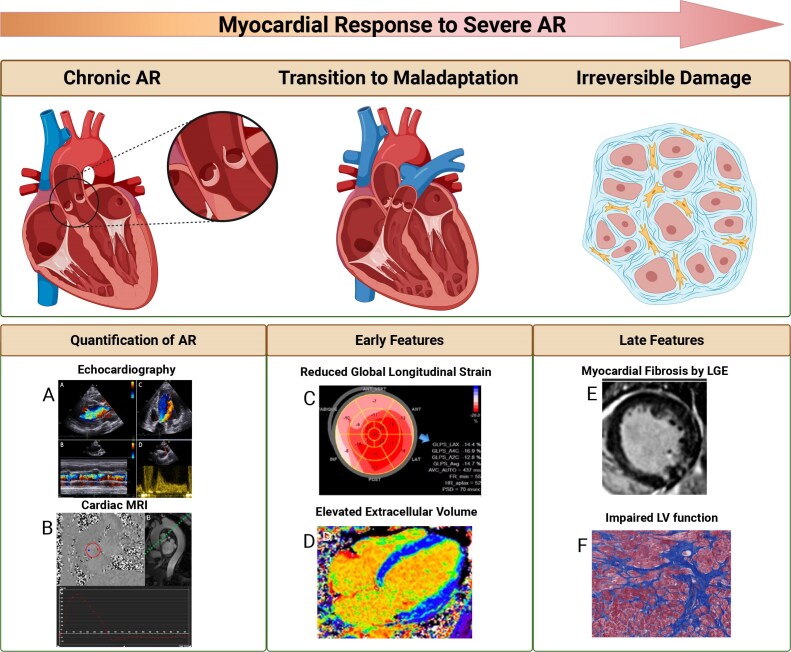
(*A*) Multipanel figure of the quantification of aortic regurgitation (AR) on echocardiography. (*A*) Parasternal long axis (PLAX) view with colour flow Doppler showing severe AR. The colour baseline can be shifted in order to measure the proximal isovelocity surface area. (*A*, *B*). M-mode with colour flow doppler transecting the AR jet in the PLAX view to measure the proportion of the left ventricular outflow tract filled by the AR jet. (*A*, *C*). Apical 5 chamber view demonstrating jet of severe AR (*a*, *d*). Pulsed wave Doppler measured in the proximal descending aorta showing holodiastolic flow reversal. (*B*). Multipanel figure of the assessment of AR and myocardial remodelling by CMR. (*B, A*). Two-dimensional phase contrast imaging of the aortic arch at the level of the main pulmonary artery. Flow is measured in the descending aorta by drawing a contour in all phases. (*B, B*). Parasagittal balanced steady state free precession image of the aorta. Flow plane in (B, *A*). marked in red. (*b*, *c*). Flow profile in the descending aorta, showing holodiastolic flow reversal. (*C*) Strain imaging demonstrating global reduced global longitudinal strain. (*D*). Four-chamber extracellular volume (ECV) map showing patchy increase in ECV throughout the myocardium. (*E*). Late gadolinium enhancement image showing patchy non-infarct pattern scar throughout the myocardium (particularly in the inferior wall and septum). (*F*). Septal myocardial biopsy (unpublished data from our institution) from a patient with severe AR stained with Masson’s trichrome showing islands of replacement fibrosis within the myocardium. This patient had persistent LV dilatation without significant remodeling after AVR. Created in https://BioRender.com.

Highlights

- Many patients awaiting guideline thresholds for intervention in chronic AR have irreversible myocardial scarring, which may result in worse prognosis after valve replacement.
- Advanced imaging techniques such as cardiac MRI can precisely quantify AR severity, LV size and fibrosis, identifying advanced remodelling that may be missed by two-dimensional echocardiography.
- Integrating fibrosis burden, strain and volumetric indices into decision making may allow earlier, patient-specific valve surgery, maximizing reverse remodelling and long-term outcomes.

## Introduction

Chronic aortic regurgitation (AR) is a common valvular pathology that imposes chronic volume and pressure overload on the left ventricle (LV), often manifesting in mid-life (30s–60s) in bicuspid aortic valve disease and in later life with tricuspid aortic valve disease.^1–3^

Eccentric hypertrophy and chamber dilation develop to accommodate the regurgitant volume, allowing the LV to maintain stroke volume for years or even decades with few symptoms.^4^ This prolonged asymptomatic phase of chronic AR belies ongoing structural remodelling of the myocardium. Eventually, the adaptation reaches its limits: myocardial contractility declines, fibrosis accumulates, and patients enter a phase of decompensation marked by symptoms (exertional dyspnoea, fatigue) and irreversible LV dysfunction.

Optimal timing of surgical intervention in chronic AR remains challenging. Early surgery (while asymptomatic and before significant dysfunction) can preserve LV function but carries operative risks, whereas delayed surgery risks irreversible heart failure and suboptimal recovery. Clinical guidelines^5,6^ attempt to balance these considerations by recommending surgery when specific triggers are met, even if the patient feels well. However, the traditional triggers: symptom onset, a drop in LV ejection fraction (LVEF), or marked LV enlargement on echocardiography are imperfect proxies for underlying myocardial health. There is evidence in contemporary cohorts that mortality in asymptomatic severe AR is higher (3.4% annually) than previously thought,^7^ and growing concern that current criteria may prompt intervention too late, after pathologic changes have become irreversible.^8^

In recent years, advancements in cardiac imaging and a deeper understanding of myocardial biology have shed new light on chronic AR. In particular, cardiovascular magnetic resonance (CMR) has emerged as a gold standard for quantifying regurgitant volume, chamber volumes, and myocardial fibrosis with high reproducibility.^9–11^ Late gadolinium enhancement (LGE) can detect focal replacement fibrosis (scar), while T1 mapping yields the extracellular volume (ECV) fraction, a marker of diffuse interstitial fibrosis. These techniques enable direct assessment of myocardial injury that may not be apparent based on structural assessment of volumes, mass and function alone. Early fibrosis may be the ‘missing link’ that heralds LV decompensation even when pump function is still preserved. Analogy with AS suggests this may aid the differentiation of reversible and irreversible remodelling and better characterize risk.^12–17^ As such, there is interest in using fibrosis and other imaging biomarkers to refine the timing of surgery—essentially, a shift towards myocardial biology-informed decision-making.

This article provides a comprehensive review of the assessment of chronic AR with an emphasis on myocardial fibrosis and its implications for management. We explore current guideline recommendations and their limitations and the advantages of different diagnostic imaging tools in assessment and risk stratification. Finally, we explore the pathophysiology of myocardial fibrosis and its potential role in clinical decision making.

## Current guidelines and their limitations

Both the American (ACC/AHA 2020^5^) and European (ESC/EACTS 2021^6^) guidelines emphasize symptom status, LV dimension and function thresholds as class I triggers for intervention in chronic AR. In symptomatic patients with severe AR, aortic valve surgery is unequivocally recommended (Class I) regardless of LV function. This reflects the imperative to alleviate symptoms and prevent further deterioration. The controversy lies in management of asymptomatic patients, where the goal is to intervene ‘just in time’, before irreversible damage, but not too early to subject low-risk patients to surgery needlessly.

Surgery is recommended (Class 1) when LV ejection fraction (EF) is ≤50% and considered at ≤55% by European guidelines, while the American guidelines take a more proactive stance with ≤55% as the threshold. LV chamber dilation is the second major criterion. A threshold of 50 mm for LV end-systolic dimension (LVESD) (25 mm/m^2^ when indexed) would trigger consideration of surgery by both guidelines. Additionally, extreme LV end-diastolic dimensions can trigger surgery: ESC guidelines include LV end-diastolic diameter (LVEDD) > 65–70 mm as a criterion (Class IIa) especially if surgical risk is low.

**Figure 1:**
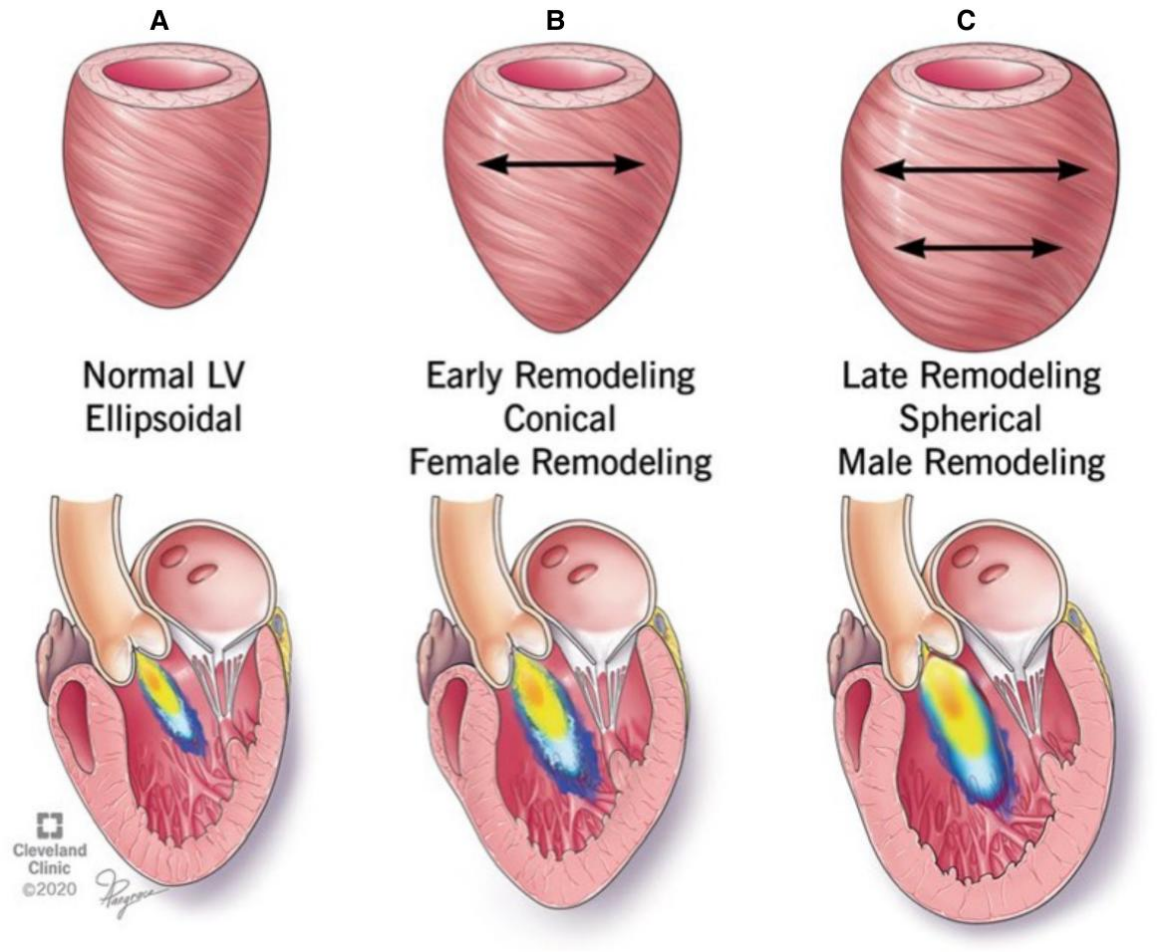
Sex-related differences in myocardial remodelling patterns in AR. Reprinted from Tower-Rader *et al*.^19^ creative commons license here http://creativecommons.org/licenses/by/4.0/. (*A*) Normal left ventricle. (*B*) Conical pattern of LV remodelling seen more commonly in women, and early in male AR remodelling. (*C*) Spherical remodelling more commonly seen in males.

While these cut-offs (EF ∼50% and LVESD ∼50 mm) have guided practice for decades, they are inherently crude metrics of myocardial health. An EF of 55% in AR can be ‘pseudo-normal’ due to the high stroke volume; subtle contractile dysfunction or rising filling pressures may be present despite a preserved EF. Likewise, a linear diameter ≥50 mm captures advanced dilation in an average or large person but fails to adjust fully for patient body size or ventricular geometry. The LV remodels spherically in some cases and more elliptically in others; a single linear dimension might underestimate true volumetric enlargement in certain geometries (*Figure 1*).^18^

Two patients with the same LVESD could have different volumes and wall stress depending on LV shape and wall thickness. Furthermore, women and smaller individuals tend to have smaller absolute LV dimensions. The ESC has acknowledged this by suggesting an indexed LVESD >25 mm/m^2^ (roughly equivalent to 50 mm in a 2 m^2^ person). Indeed, a recent study found that asymptomatic patients began to incur higher mortality once LVESD reached 20 mm/m^2^, a value below the guideline cut-off, highlighting that significant risk may already be present earlier in the remodelling process.^8^ The ‘one-size-fits-all’ nature of current triggers is increasingly problematic.

A recent analysis of 525 patients with severe AR demonstrated that the optimal LV volume threshold to predict adverse events varies markedly by age and sex.^20^ On serial follow-up, older patients and women had consistently smaller LV volumes than younger patients and men for the same severity of AR. Consequently, the LV end-systolic volume index (LVESVi) associated with impending adverse events was highest for young men (∼50 mL/m^2^), intermediate for older men (∼35 mL/m^2^), and lowest for women (∼27 mL/m^2^). In other words, women and older individuals reached their ‘danger zone’ at much smaller LV sizes. Current guidelines do not account for these differences, they apply identical (indexed) diameter cut-offs regardless of a patient’s sex or age. This raises concern that women and the elderly may undergo ‘late’ surgery, as their ventricles might never enlarge to 50 mm even when pathological remodelling is well underway.

The use of 2D echocardiographic diameters is particularly problematic here, as it is fraught with measurement variability and assumes symmetric dilation. Three-dimensional (3D) echo or CMR-derived LV volumes provide a more comprehensive assessment of remodelling, but these are not explicitly incorporated into guidelines beyond general advice to consider surgery if there is ‘severe LV enlargement’. Lastly, the triggers focus on mechanical indices (EF, dimension) and symptoms, without direct measurement of the condition of the myocardial tissue. It is now evident that myocardial fibrosis can accrue silently during the asymptomatic phase and may lead to a decline in LV function.^21^ By the time EF falls below 50% or the patient develops overt symptoms, fibrosis may have already irreversibly impaired LV compliance and contractility.^4^ The guidelines do acknowledge the rationale: they aim to intervene ‘before the irreversible long-term consequences of volume overload’. Yet in practice, waiting for EF to drop or dimensions to cross a fixed threshold may allow fibrosis, the key irreversible consequence, to take hold.

## LV remodelling in chronic AR: from compensation to maladaptation

Chronic AR sets in motion LV remodelling that can be broadly divided into an initial adaptive phase and a later maladaptive phase (*Graphical Abstract*). In the adaptive phase, the LV undergoes eccentric hypertrophy: new sarcomeres are added in series, enlarging chamber size while relatively preserving wall thickness ratio. This allows the LV to accommodate the high end-diastolic volume (EDV) without a drastic rise in end-diastolic pressure.^22,23^ Stroke volume increases to maintain forward output despite a portion regurgitating backward. As a result, patients often remain asymptomatic for years with normal exercise tolerance. During this phase, LVEF is typically normal or supranormal (≥60%), because the Frank-Starling mechanism and enhanced preload lead to vigorous contraction. Importantly, even though EF is normal, the LV is working hard, the total stroke volume is elevated, and myocardial oxygen demand is increased. Still, as long as compensatory mechanisms suffice, heart failure symptoms are absent and conventional metrics appear reassuring.

However, chronic volume and pressure overload eventually drive the LV into a maladaptive phase. The myocardium can no longer keep up with the haemodynamic stress by dilation alone. Wall stress increases, particularly systolic wall stress due to afterload from the regurgitant volume and any concomitant hypertension.^24,25^ This triggers further hypertrophy (adding sarcomeres in parallel, increasing wall thickness) in an attempt to normalize wall stress, often resulting in an enlarged but thick-walled LV.^22^ Over time, myocyte injury and death can occur from energetic supply-demand mismatch and wall stress, leading to fibrosis.^23^ The biochemical milieu in volume overload (stretch-mediated signalling, TGF-β activation, Angiotensin II, aldosterone, etc) stimulates cardiac fibroblasts to produce collagen.^26,27^ Myocardial histopathology studies in AR have shown increased collagen deposition in the extracellular matrix in patients with chronic AR.^17,27–32^

The transition from reversible myocyte hypertrophy to irreversible cellular damage is gradual and insidious. Diffuse interstitial fibrosis begins to accumulate in the myocardium, initially as a response to chronic stretch, neurohormonal activation (renin-angiotensin-aldosterone system stimulation), and possibly subendocardial ischemia due to reduced diastolic coronary perfusion pressure in AR.^24,25^ This interstitial fibrosis stiffens the ventricle (raising filling pressures) and subtly impairs contractility, even before EF falls. Eventually, replacement fibrosis (scar) may form.

Focal fibrosis is essentially the point of no return in remodelling, whereas hypertrophy regresses after valve correction, scar tissue does not. As fibrotic remodelling progresses, the LV loses its compliance and contractile reserve. Patients may then develop diastolic dysfunction (impaired filling, higher pulmonary pressures) and later systolic dysfunction. The classic tipping point of decompensation in AR is when EF begins to decline from its previously maintained level, signalling that the ventricle can no longer compensate for the volume overload.^30^ This often correlates with patients developing symptoms such as exertional dyspnoea, reduced exercise capacity, or fatigue. At this stage, LV end-systolic volume has typically increased (a marker of contractile dysfunction), and the risk of heart failure and death rises.^8,33^

## Imaging modalities for AR: beyond 2D echocardiography

Transthoracic echocardiography remains the first-line imaging modality in evaluating chronic AR. TTE provides a comprehensive assessment: it identifies the cause of AR (valve morphology, e.g. bicuspid valve, prolapse, degeneration), quantifies AR severity (via colour Doppler jet, vena contracta, regurgitant volume and fraction), and evaluates the LV’s response to volume load (dimensions, wall thickness, EF). The strengths of echocardiography are its wide availability, non-invasiveness, and ability to track changes over time. Guideline trigger measurements (EF and LVESD/LVEDD) are conventionally obtained by 2D TTE, usually from parasternal long-axis linear dimensions or biplane Simpson’s method for volumes.^6^

However, 2D echocardiographic measurements have important limitations in AR. Linear dimensions are single-axis measurements that may not reflect true 3D chamber enlargement.^34,35^ 2D Simpson’s volumetric EF on echo can be more representative, but in dilated ventricles endocardial border tracing may be challenging, especially if acoustic windows are poor. Measurement variability is non-negligible, small errors in calliper placement can swing a patient above or below a surgical threshold. To improve accuracy, 3D echocardiography has been increasingly applied in valvular disease.^36^ Three-dimensional echo can directly measure LV end-diastolic and end-systolic volumes without geometric assumptions, and it avoids the foreshortening that can occur in 2D views. In AR, 3D TTE has shown better reproducibility for volume quantification compared with 2D, and it correlates more closely with CMR (the reference standard) for LV volumes. This suggests that 3D echo could detect earlier volume overload changes and more reliably track remodeling.^36^ Despite its promise, 3D echo is underutilized and is not yet central in guidelines, partly due to limited outcome data.^37^

Global Longitudinal Strain (GLS) by echo is another marker that can unmask subclinical LV dysfunction. GLS (measured by speckle-tracking echocardiography) quantifies myocardial deformation and typically becomes abnormal (less negative) before EF drops. In chronic AR, GLS may decrease even while EF is still preserved, indicating early systolic dysfunction. Depressed GLS has been associated with impending LV functional decline.^37^ Although there is not a firm guideline threshold, an abnormal GLS can raise concern that the ‘true’ LV function is worse than the EF suggests (EF in AR can be misleadingly maintained by high preload). Thus, GLS can be a useful adjunct in timing decisions for asymptomatic AR, a significantly reduced strain (less negative than −18% for example) might tip the scales towards earlier surgery in an equivocal case.

### Cardiovascular magnetic resonance

CMR has become an invaluable tool in AR evaluation, often considered the non-invasive reference standard for volume and fibrosis assessment. CMR offers several advantages including precise chamber and AR quantification, and assessment of myocardial fibrosis.^9,11,38^

### CMR for LV volumes and AR quantification

**Figure 2:**
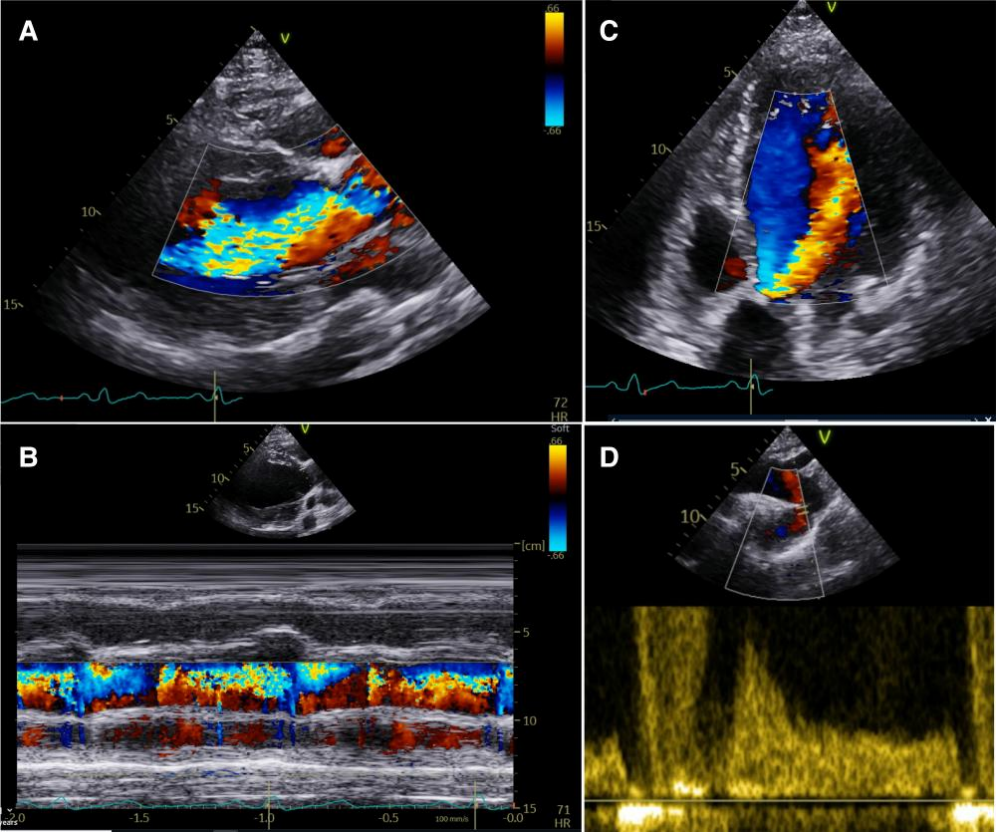
Echocardiography for AR quantification. (*A*) PLAX view with colour flow Doppler showing severe AR. The colour baseline can be shifted in order to measure the proximal isovelocity surface area. (*B*) M-mode with colour flow Doppler transecting the AR jet in the PLAX view to measure the proportion of the left ventricular outflow tract filled by the AR jet. (*C*) Apical 5 chamber view demonstrating jet of severe AR. (*D*) Pulsed wave Doppler measured in the proximal descending aorta showing holodiastolic flow reversal.

**Figure 3:**
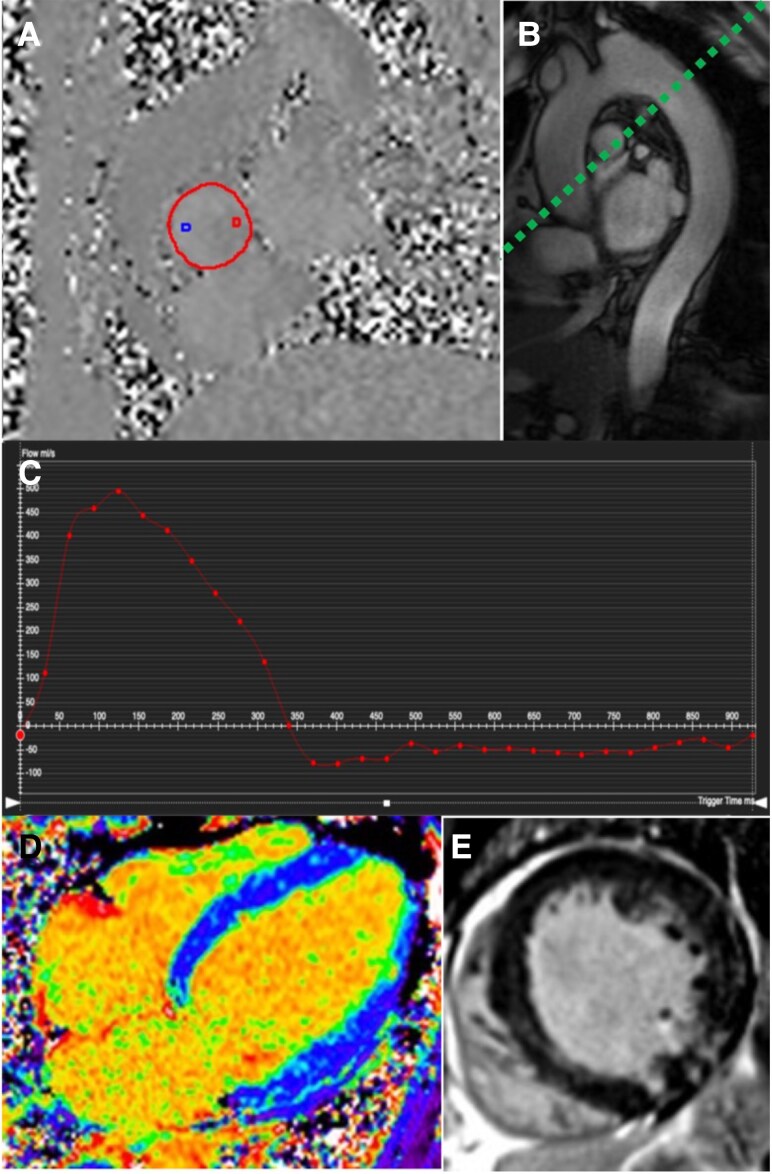
Assessment of AR and myocardial remodelling by CMR. (*A*) Two-dimensional phase contrast imaging of the proximal ascending aorta just above the aortic valve. Flow is measured by drawing a contour (red) in all phases. (*B*) Parasagittal balanced steady state free precession image of the aorta- visual assessment of flow reversal can be performed. A flow plane could be planned (green dotted line) to quantify flow reversal in the descending aorta. (*C*) aortic regurgitation. (*D*) Four-chamber ECV map showing patchy increase in ECV throughout the myocardium. (*E*) LGE image showing patchy non-infarct pattern scar throughout the myocardium (particularly in the inferior wall and septum).

CMR cine imaging (steady-state free precession sequences) can directly measure LV end-diastolic and end-systolic volumes without geometric assumptions, yielding highly accurate EF and chamber size measurements. CMR offers a direct approach to AR quantification by measurement of regurgitant fraction (RF) through 2D phase contrast imaging,^39^ making it a cornerstone to accurate AR severity assessment, where echocardiography quantification is uncertain (*Figure 2*). In the absence of shunts and significant mitral and tricuspid regurgitation, comparison of RV and LV stroke volumes offers internal validation and makes CMR a robust technique for regurgitant volume quantification (*Figure 3*).^10^ This is important as flow measurement by CMR is not without its own limitations, due to turbulent blood flow, particularly important in the presence of mixed aortic valve disease, concomitant aortic pathology, eccentric AR jets and arrhythmia.^11^

Studies have shown CMR-derived LV volumes and regurgitant fractions have prognostic significance. CMR-assessed LVESVi and RF can stratify risk among asymptomatic patients: one multicentre study found that an LVESVi ≥45 mL/m^2^ and AR fraction ≥32% identified asymptomatic patients at higher risk of death or heart failure admission.^40^ A recent multicentre registry study found a RF of 43% to be associated with development of guideline indications for surgery, differing from previous studies, and this was attributed to differences in study populations, who may have differing underlying myocardial substrate and response to a given RF.^41^ This difference highlights that the thresholds determined in observational studies may be subject to selection bias and further research is required to define more precise thresholds for ‘clinically significant AR’. A randomized controlled trial of CMR guided intervention is required in this area. Regardless, it is apparent that these thresholds are lower than those determined by echocardiography and likely require their own reference ranges.^42,43^

### Myocardial fibrosis assessment in AR

Myocardial fibrosis has emerged as a critical determinant of outcomes in patients with chronic AR. Intuitively, a ventricle that has developed significant fibrosis has less reversible remodelling and a higher likelihood of post-surgical dysfunction.^30^ A growing body of evidence from CMR studies supports this, linking both focal scar (replacement fibrosis) and diffuse fibrosis to adverse clinical endpoints.

LGE imaging can directly visualize focal myocardial fibrosis (replacement fibrosis). After injection of gadolinium contrast, scarred myocardium retains gadolinium longer (due to increased extracellular space and delayed washout), appearing as hyper-enhanced regions on T1-weighted images. In AR patients, LGE may appear in a mid-wall pattern (suggesting non-ischemic fibrosis in the interstitial space of the mid-myocardium, often in the septum or ventricular free wall).^4^ The prevalence of LGE in severe AR varies by cohort, but a sizeable minority have detectable scar. For example, one study reported LGE in approximately one-third of patients (33%) with moderate or severe AR, most of which (90%) was a non-ischemic pattern.^21^

Beyond focal scar, CMR T1 mapping allows quantification of diffuse interstitial fibrosis via the extracellular volume fraction (ECV%). By measuring myocardial T1 relaxation times before and after gadolinium and adjusting for blood pool T1, one can derive the fraction of myocardium that is extracellular space.^44^ In chronic AR, CMR studies have shown ECV% values mildly to moderately elevated compared with normal controls, even in asymptomatic patients.^16^ A particularly useful metric is the indexed extracellular volume (iECVol), which is the absolute volume of extracellular matrix in the LV, indexed to body surface area (essentially ECV% multiplied by LV myocardial volume). As the LV dilates in AR, iECVol can increase substantially even if ECV% is only modestly increased, reflecting the combined effect of a bigger heart and more fibrosis. Indeed, one CMR study found that iECVol had a strong association with AR severity and outcomes, whereas ECV% and LGE scar did not significantly correlate with AR grade.^45^

The presence of any LGE scar in chronic AR has been linked to worse outcomes. A pivotal study of nearly 400 patients by Malahfji *et al*.^21^ showed that patients with myocardial scar (either infarct or mid-wall) had over 3.5-fold higher unadjusted mortality risk, and scar remained an independent predictor of all-cause mortality with a hazard ratio ∼2.5 even after adjusting for EF, age, and other factors.^21^ Notably, in that study, LGE was a stronger predictor of death than the traditional guideline triggers of EF < 50% or LVESD >50 mm. This suggests that scar is capturing risk that EF and dimension criteria might miss. Importantly, patients with scar who underwent intervention had a significantly lower mortality than those who did not, implying that surgery mitigated some of the scar-related risk. In other words, identifying scar could identify patients who would benefit from ‘early’ surgery.

Diffuse fibrosis measured by CMR has prognostic value as well. Senapati et al. reported that an iECVol ≥24 mL/m^2^ in patients with AR (in combination with RF ≥30%) portended the highest risk of death or need for surgery, defining a high-risk cohort.^45^ Importantly, iECVol rose progressively with CMR regurgitant fraction, becoming significantly higher at the conventionally considered moderate range (RF >30%) and it was the combination of these factors that was important. Patients with large RF but low fibrosis burden fared better than those with comparable AR severity but high iECVol, indicating fibrosis burden helps differentiate maladaptive remodelling.

Diffuse interstitial fibrosis (ECV) measured by CMR correlates with symptom burden in chronic AR, with higher ECV linked to dyspnoea and reduced exercise capacity even when LV size is similar. Moreover, women exhibit rising ECV in proportion to regurgitant volume and become symptomatic at lower LV volumes, whereas men show no significant ECV change, suggesting that earlier fibrotic stiffening may drive the earlier onset of symptoms in women.^46^

## Factors affecting recovery after AVR

Reduction of LV preload and afterload by AVR commonly results in reverse remodelling with reductions in LV volumes and mass. This occurs promptly within the early post-operative period and continues to improve up to 1 year and beyond.^47^ A retrospective analysis of 172 adult patients who underwent AVR for severe AR showed that 65% patients achieved LV size and function normalization after surgery (though 1/3 underwent surgery before meeting guideline indications). Elevated presurgical LV ESD was associated with lack of LV normalization (best cut-off 43 mm) and was associated (along with LV ESD) with adverse outcomes at up to 10 years follow-up.^48^

Another study evaluated pre- and post-operative changes in 29 patients with severe AR and 59 patients with severe mitral regurgitation, taking advantage of the precision volumetric assessment of CMR.^49^ The degree of dilatation was greater for a given regurgitant volume in AR. There was a reduction in LV volumes in both MR and AR, but with residual elevated LV mass compared with controls in the AR group at a median of 7 months after AVR. The only predictor of incomplete reverse remodelling was pre-operative LV EDVi, highlighting the potential value of CMR in pre-operative risk assessment. An LV EDVi of 155 mL/m^2^ was found to be associated with incomplete regression.

### Fibrosis and reverse remodelling

A study of 32 patients with severe AR and 67 with severe AS found a reduction in ECVol in both groups but more in the AR group than the AS group with stable ECV%, suggesting balanced regression of cellular and extracellular components and significant plasticity of diffuse fibrosis. This suggests that diffuse interstitial fibrosis can regress once the volume overload is relieved. The window for reversibility is not indefinite; operate too late and diffuse fibrosis transitions into irreversible scar.^50^

Collectively, these findings advocate for integrating myocardial fibrosis assessment into the management of AR. By identifying high-risk patients (those with fibrosis) earlier, clinicians might refer them for surgery at a stage when outcomes can be improved. Conversely, an asymptomatic patient with severe AR but no evidence of fibrosis and otherwise low-risk features might be safely observed a bit longer with close monitoring. The next section will explore into how patient-specific factors can influence remodelling and fibrosis, which further supports moving away from blanket criteria toward a personalized approach.

## Influence of age, sex, and comorbidities on remodelling and fibrosis

Chronic AR does not affect all patients uniformly. There are noteworthy differences in how the LV adapts to AR depending on patient-specific factors like age, sex, and the presence of other cardiovascular conditions. Appreciating these differences is important, because it means some patients may reach a critical point for surgery sooner (or later) than others, even if the absolute AR severity is the same.

### Sex differences

Men and women exhibit different remodelling patterns in response to volume overload. Women generally have smaller ventricles to begin with, and studies suggest they may develop proportionally less LV dilation but more fibrosis and symptoms at smaller thresholds. In a recent study it was shown that women had lower absolute LV volumes and mass than men across all degrees of AR severity.^46^ After indexing for body size, EDV was similar, but men had larger end-systolic volume and slightly lower EF, indicating men’s hearts dilated more before losing systolic function. Crucially, women were more likely to report symptoms (NYHA class II or higher) than men despite similar AR grades, yet they underwent surgery at similar rates. ECV% (diffuse fibrosis) increased with regurgitant volume in women, but not in men. In other words, women’s myocardium showed increasing fibrotic remodelling as AR got more severe, whereas men’s did not show a significant ECV% change. This difference in myocardial response might contribute to women developing stiffness and symptoms sooner. It also suggests that a woman with severe AR might have significant fibrosis even if her LV dimensions haven’t exceeded guideline cutoffs, putting her at risk if one waits for the same numeric triggers as in men.

### Effect of age

The myocardium’s adaptability also changes with age. Younger patients (e.g. in 20 s–40 s) with chronic AR often tolerate larger degrees of dilation with maintained EF, their myocardium is more compliant and can hypertrophy more easily. Older patients are more prone to diastolic dysfunction and have stiffer ventricles (often some degree of age-related fibrosis or hypertension-related remodelling). In a 2023 study by Akintoye *et al*.^20^ older patients (≥60 years) had significantly smaller LV volumes at baseline than younger patients for severe AR (mean LVESVi 27 vs. 32 mL/m^2^).^20^ Older hearts ‘decompensate’ earlier in terms of volume burden. This is likely because of concomitant comorbidities like hypertension and intrinsic myocardial stiffness (fibrosis) that comes with aging.

In clinical practice, these factors mean that managing AR should be individualized. A young athletic man with bicuspid AR might tolerate an LVESD of 50 mm without symptoms, but a 60-year-old woman with the same LVESD might already be short of breath, and the latter may have more fibrosis underlying. Recognizing patient heterogeneity is thus an argument against rigidly applying uniform cutoffs and for incorporating broader assessments (symptoms, exercise testing, biomarkers, imaging findings) into the decision.

## Toward myocardial biology-informed timing of surgery: clinical implications

The collective insights from advanced imaging and patient-specific factors call for a more nuanced, ‘biology-informed’ approach to the timing of aortic valve surgery in chronic AR. Rather than relying solely on 2D echocardiographic measurements and symptomatic status, clinicians are increasingly urged to consider underlying myocardial biology, specifically, the presence of fibrosis and subtle functional changes, in decision making. In essence, there is mounting evidence that we should consider intervention before irreversible myocardial damage has occurred, even if traditional triggers are not yet met.

In patients with severe AR who remain ‘in the gray zone’ by conventional criteria, additional markers could guide earlier surgery: CMR-detected fibrosis (mid-wall LGE or elevated ECV%/iECVol) signals myocardial injury^21^; serial declines in EF or reduced GLS reveal subclinical systolic dysfunction^51^; an LVESVi ≥45 mL/m^2^ on 3D echo or CMR denotes high-risk remodeling^40^; rising natriuretic peptides (e.g. BNP ≥130 pg/mL) indicate escalating wall stress^52^; and exercise testing that provokes symptoms unmasks latent functional impairment.^5^ Integrating these imaging and functional biomarkers within a multidisciplinary Heart Team framework enables personalized timing of AVR, guided by the myocardial response to volume overload, allowing intervention before irreversible myocardial injury occurs.

The presence of fibrosis (scar or diffuse fibrosis) shifts the risk-benefit toward earlier surgery, because continuing to wait likely yields diminishing returns (the ventricle won’t recover what’s lost and might lose more). Conversely, if a patient has zero fibrosis, normal strain, normal natriuretic peptides, and is asymptomatic, one could justify watchful waiting even if they are close to a threshold, with frequent follow-up.

**Figure 4:**
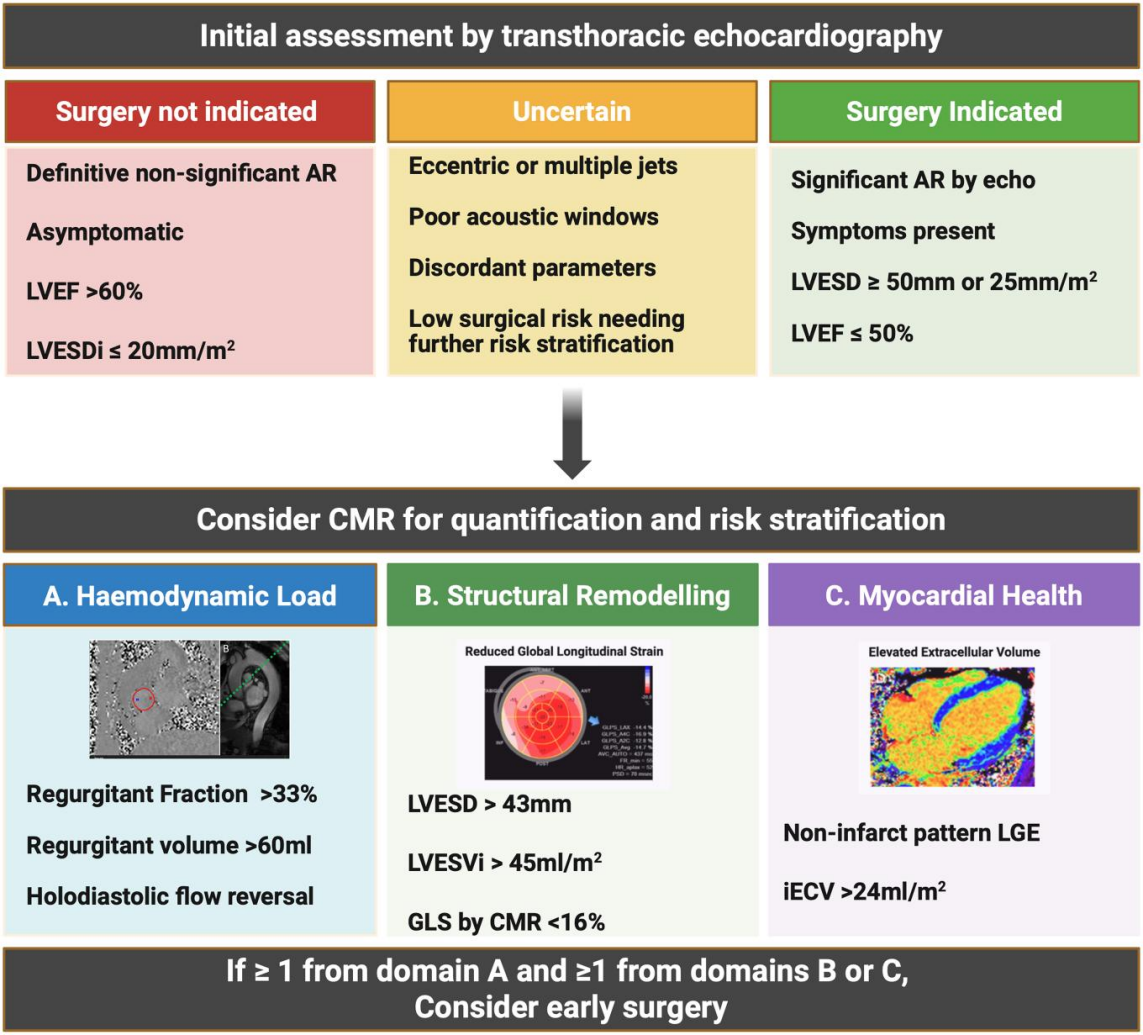
Algorithm for incorporating CMR into the assessment for early surgery in patients with AR where transthoracic echocardiography is uncertain. (*A*) Two-dimensional phase contrast imaging of the proximal ascending aorta just above the aortic valve. Flow is measured by drawing a contour (red) in all phases. Parasagittal balanced steady state free precession image of the aorta- visual assessment of flow reversal can be performed. A flow plane could be planned (green dotted line) to quantify flow reversal in the descending aorta. (*B*) 17 segment left ventricular bulls eye plot demonstrating a pattern of reduced GLS. (*C*) Four-chamber ECV map showing patchy increase in ECV throughout the myocardium.

An integrated approach means using the full armamentarium of diagnostic tools to decide when to intervene: not just how the patient feels and 2D echo, but also CMR fibrosis/volume metrics, exercise capacity, biomarkers, and individualized considerations. CMR in particular has a role in risk stratification for early surgery where traditional echocardiographic assessment is uncertain. (*Figure 4*). It moves us toward personalized medicine in valvular heart disease. The expected outcome is to improve long-term results, maximizing the chance that surgery is done while the LV can still recover fully, and minimizing instances of irreversible LV dysfunction or late referral.

## Future directions

While substantial progress has been made in understanding chronic AR and its impact on the myocardium, several important questions remain. Future research and clinical innovation will be needed to fully realize a paradigm shift toward biology-informed management. To date, most evidence linking fibrosis to outcomes in AR is observational. The logical next step is a prospective trial or registry to test fibrosis-guided surgical thresholds.

The future of AR management will also be influenced by improvements in surgical and transcatheter techniques. Valve repair in select AR patients can preserve the native valve and avoid prosthetic complications; if repair techniques become more widely applicable with durable results, surgeons may be more inclined to operate earlier since the downsides of a prosthetic valve (lifelong anticoagulation, etc.) are avoided.^53^

Transcatheter aortic valve implantation (TAVI) for AR is still in its infancy (AR has no calcification to anchor the valve and often an enlarged annulus). Newer generation TAVI devices and dedicated AR devices are being trialled. If TAVI for AR becomes safe and effective, the threshold for intervening early might lower, especially in older high-risk surgical patients, because the procedure risk would be less. Ongoing studies of TAVI in pure AR (using devices like the J-Valve or JenaValve) will be important.^54^

## Conclusion

Management of chronic AR is gradually shifting from reliance on simple dimensional and functional triggers to a more nuanced consideration of myocardial health. Although observational studies highlight myocardial fibrosis as an early marker of irreversible injury and suggest that imaging biomarkers may refine timing of surgery, prospective trials are still needed to confirm that a fibrosis-guided approach improves outcomes. In the meantime, integrating advanced imaging (CMR fibrosis and volumetrics, strain analysis), biomarkers and patient-specific factors within a multidisciplinary Heart Team can help tailor intervention and potentially preserve ventricular function. Ultimately, the goal remains to intervene at the stage when valve replacement or repair offers the best chance of restoring and maintaining myocardial integrity, while acknowledging that the optimal thresholds for such biology-driven decisions await definitive clinical trial evidence.

## Data Availability

No new data were generated or analysed in support of this research.
